# Lessons learnt from a measles outbreak in Madang Province, Papua New Guinea, June 2014 – March 2015

**DOI:** 10.5365/WPSAR.2016.7.2.013

**Published:** 2017-03-14

**Authors:** Karoi Kamac, Beverley Paterson, James Flint

**Affiliations:** a Provincial Health Office, Madang Province, Papua New Guinea.; b University of Newcastle, NSW, Australia.; c Hunter New England Health, NSW, Australia.

## Abstract

**Objective:**

This study examined measles vaccine wastage during an outbreak response in Madang Province of Papua New Guinea from June 2014 to March 2015.

**Methods:**

Vaccine wastage was defined as the number of doses received by a health centre minus the total number of doses administered during and returned following the outbreak vaccination campaign. Vaccine data were collected from the Provincial Health Information Office, the Provincial Vaccine Store register and clinic and health centre immunization registers for calculating the vaccine wastage. Interviews were conducted with all 48 health centres involved in the outbreak response using a structured questionnaire to explore the reasons for vaccine wastage.

**Results:**

Of the 154 110 doses issued by Madang Province during the outbreak, a total of 85 236 (55%) doses were wasted. The wastage varied by district from 31% to 90%. The total cost of the vaccine wastage was estimated to be 589 810 Kina (US$ 196 604). None of the health centres maintained vaccine stock registers. Most health centres indicated multiple failures in cold chain logistics. Almost 40% of health centres reported incorrectly diluting vaccines. The same percentage of health centres reported using incorrect injection techniques.

**Discussion:**

Regular audits of cold chain logistics, staff training and improved processes for recording vaccine administration and wastage will decrease vaccine wastage during vaccine-preventable disease outbreaks and also benefit routine immunization activities.

## Introduction

A measles outbreak in Papua New Guinea affected all 22 provinces, spanned nine months from June 2014 to March 2015 and resulted in a reported total of 11 097 cases. (*1*) In Madang Province there were 5073 measles cases and 30 deaths recorded. (*1*) During 2009–2013, Madang Province had an average reported measles vaccination coverage of 38%. (*2*) A large-scale national vaccination campaign was implemented to bring the outbreak under control; in Madang Province, the campaign went from 1 June 2014 to 31 March 2015. During this campaign, 2.7 million doses of measles vaccine were supplied to all provinces by the National Expanded Program on Immunization (EPI) unit.

The World Health Organization (WHO) estimates that over 50% of vaccine doses administered during routine immunization programmes are wasted around the world. (*3*) These high wastage rates are a key factor driving up costs of the EPI. This paper reports on measles vaccine wastage and the reasons for this wastage during the 2014–2015 measles outbreak in Madang Province, Papua New Guinea.

## Methods

All 48 health centres (front-line health clinics that serve as the base for vaccination programmes, including mobile and outbreak clinics) in all six districts of Madang Province were included in this retrospective cross-sectional study of measles vaccine wastage during the outbreak. As there were no measles vaccines in any of the health centres before the vaccination campaign (due to an extended stock-out of measles vaccine), no vaccines were returned to the provincial office following the campaign, and the number of doses left in health centres after the campaign was assumed to be small. The start and end balances of the vaccine doses were not counted in calculating vaccine wastage rate. The vaccine wastage rate during the campaign was calculated using the following formula: 1 – (number of doses administered/number of doses issued) × 100%. Data for the study were collected from the Provincial Health Information Office, the Provincial Vaccine Store register, clinic and health centre immunization registers and through interviews with 48 team leaders (one from each of the 48 health centres) who coordinated the vaccination response during the outbreak. Telephone interviews were conducted using a structured questionnaire that captured information on the knowledge, skills and techniques used in vaccine management. A retrospective review of vaccine practices during the outbreak was also conducted by discussions with the team leaders. All data were collected by the Provincial Disease Surveillance and Disaster Response Coordinator of Madang Province. The study period was from May to August 2015. All data were recorded, cleaned and analysed using Microsoft Excel.

## Results

Of the 154 110 doses issued by Madang Province during the outbreak response, a total of 85 236 (55%) doses were wasted. The wastage varied by district from 31% in Rai Coast to 90% in Middle Ramu (**Table 1**). The total cost of the vaccine wastage was estimated to be 589 810 Kina (US$ 196 604).

**Table 1:** Number of measles vaccine doses issued, wasted, percentage wasted and cost by districts in Madang Province, Papua New Guinea, June 2014 to March 2015

District	Number of doses issued	Number of doses administered	Number of doses wasted	Percentage of doses wasted	Cost of wastage (Kina)	Cost of wastage (US$)
Middle Ramu	28 260	2743	25 517	90%	164 329	54 776
Bogia	23 400	5123	18 277	78%	134 447	44 816
Sumkar	18 350	4142	14 208	77%	117 710	39 237
Madang	50 480	32 202	18 278	36%	91 499	30 500
Usino Bundi	20 950	14 055	6895	33%	44 403	14 801
Rai Coast	19 020	13 209	5811	31%	37 422	12 474
Total	160 460	71 474	88 986	55%	589 810	196 604

1 Kina = 0.32 US$

**Table 2** shows the results for the vaccine management interviews with the team leaders. None of the 48 health centres maintained vaccine stock registers. Most health centres indicated multiple failures in cold chain logistics. One third of health centres in the province did not have a functioning refrigerator. In Rai Coast district, 63% did not have functioning refrigerators. Less than half of health centres in the province had functional thermometers (44%), ice packs (42%) or cold Boxes (44%); only 44% of staff in the health centres examined vaccine vial monitors before use. Although functioning thermometers were available in 44% of the health centres, none of the outbreak teams reported using a thermometer to monitor vaccine temperatures when working in the field. For health centres without vaccine cold boxes or vaccine carriers, the vaccines were stored in borrowed cold boxes or in the cartons used to deliver the vaccines. All reconstituted vaccines were discarded at the end of each session as per WHO guidelines.

**Table 2:** Capacity for vaccine management by health centres (*n* = 48) in each district in Madang Province, Papua New Guinea, June 2014 to March 2015

Capacity	Middle Ramu (*n* = 8)	Bogia (*n* = 8)	Madang (*n* = 8)	Usino Bundi (*n* = 8)	Rai Coast (*n* = 8)	Sumkar (*n* = 8)	PROVINCE (*n* = 48)
Maintained vaccine stock register	0 (0%)	0 (0%)	0 (0%)	0 (0%)	0 (0%)	0 (0%)	0 (0%)
Functioning thermometer	4 (50%)	2 (25%)	5 (63%)	2 (25%)	3 (38%)	5 (63%)	21 (44%)
Ice packs for vaccine storage	2 (25%)	3 (38%)	5 (63%)	3 (38%)	2 (25%)	5 (63%)	20 (42%)
Maintained temperature chart	4 (50%)	2 (25%)	5 (63%)	2 (25%)	3 (38%)	5 (63%)	21 (44%)
Cold box for vaccine transport*	3 (38%)	3 (38%)	5 (63%)	3 (38%)	2 (25%)	5 (63%)	21 (44%)
Monitored vaccine vial monitors	3 (38%)	3 (38%)	5 (63%)	3 (38%)	2 (25%)	5 (63%)	21 (44%)
Functioning vaccine fridge	4 (50%)	6 (75%)	8 (100%)	5 (63%)	3 (38%)	6 (75%)	32 (67%)
Vaccine carriers**	4 (50%)	6 (75%)	8 (100%)	5 (63%)	3 (38%)	6 (75%)	32 (67%)
Correct dilution of vaccine	4 (50%)	5 (63%)	5 (63%)	5 (63%)	5 (63%)	5 (63%)	29 (60%)
Conducted small clinic sessions	5 (63%)	4 (50%)	2 (25%)	3 (38%)	3 (38%)	3 (38%)	20 (42%)
Correct injection techniques	4 (50%)	5 (63%)	5 (63%)	5 (63%)	5 (63%)	5 (63%)	29 (60%)

* Large cold box for vaccines: ~16 icepacks used to keep vaccines cool for up to 5–7 days

** Small cold box for vaccines: ~4 icepacks used to keep vaccines cool for 2–3 days

Retrospective review of vaccine practices during the outbreak indicated that 40% of teams were incorrectly preparing the vaccines (diluting with 2.5 mL of diluent instead of 5 mL) (**Table 2**). This wastage was due to a change in the size of the diluent vials and health workers being unaware of this change. Forty per cent of the teams reported vaccinators who were incorrectly using syringes, resulting in the frequent locking of syringes and discarding of vaccine.

The health centres in Madang District reported the best overall results with regards to cold chain logistics, and they also reported below average vaccine wastage (36% wastage). The two districts with the highest levels of vaccine wastage (90% for Middle Ramu and 89% for Bogia) reported the highest number of health centres conducting small clinic sessions (63% for Middle Ramu and 50% for Bogia, respectively). Only half of the health centres in Middle Ramu reported having vaccine carriers, correctly diluting vaccine and using correct injection technique.

## Discussion

This study documented the number of vaccines wasted and explored the reasons for this wastage during a measles outbreak response in Madang Province, Papua New Guinea. The cost associated with vaccine wastage was almost US$ 200 000. This estimated cost was for one antigen during one outbreak in one province. This review highlighted several areas that need to be addressed to reduce vaccine wastage during future outbreak response activities. Even though wastage during routine vaccination programmes was not evaluated in this study, efforts made to address outbreak-associated wastage will also benefit routine vaccination programmes. Investments made to reduce wastage will have significant benefits and are cost-saving in the long term; for example, the cost to replace or repair refrigerators in all facilitates in Madang Province was estimated to be less than US$ 70 000 (~35% the cost of the wasted vaccines).

India has set a routine vaccine wastage rate for most vaccines at 25%. (*4*) This Indian policy encourages opening a multidose vial for a single beneficiary to avoid any missed opportunities. WHO recommends the following wastage rates for estimating vaccine needs for routine programmes: 50% wastage for 10–20 dose vials (lyophilized vaccines) and 10% wastage for 2–6 dose vials (lyophilized vaccines). (*5*) Wastage of measles vaccine during outbreak campaigns and supplemental immunization activities (SIAs) is typically much less than during routine vaccination programmes because more children can get vaccinated in the same session. In Africa, most measles SIAs report wastage rates lower than 10%, and WHO suggests using a conservative 15% during SIA planning using 10-dose measles vaccines. (*6*, *7*) In Papua New Guinea during the measles campaign, 10-dose vials of lyophilized measles vaccines were used along with 2.5 mL diluent vials; each dose should be diluted with 5 mL of diluent. The overall wastage of 55% in this study is much higher than the 15% WHO benchmark. The high wastage was primarily due to poor cold chain logistics and incorrect vaccine preparation and administration.

This study identified an urgent need for training and supervision of health-care workers before and during SIAs, especially when there are new immunization protocols being implemented. The change in volume of the diluent vials provided by the national office, from a 5 mL diluent vial to a 2.5 mL diluent vial, resulted in double strength vaccines being administered. Health-care workers were familiar with using 5 mL diluent vials and failed to realize the need for using two 2.5 mL vials per dose during the campaign. Almost 40% of health centres reported incorrectly diluting the vaccine during the outbreak response. There is also an urgent need for the national and/or provincial immunization programmes to review the vaccine logistics and procurement processes which led to incorrect diluent vials (2.5 mL instead of 5 mL) being bundled with the measles vaccine during this campaign. Clear instructions from the national/provincial levels on the use of the 2.5 mL vials were not adequately issued or conveyed to the field staff. Also, adequate training and supervision on the use of auto-disable syringes was not provided to field staff. Auto-disable syringes prevent the administration of vaccine if incorrect techniques are used. This safety feature results in high levels of wastage when poor injection techniques are employed. During the measles campaign, newly graduated health-care workers who had not used the auto-disable syringes were recruited. Training of staff on correct injection techniques should be provided on a regular basis and especially when new staff are employed for SIA or routine vaccination programmes.

Poor documentation and communication resulted in the indiscriminate dissemination of vaccines to health centres and poorly planned clinics. Improper recording and reporting of vaccine stocks and not knowing the target population size during field clinics often resulted in a large number of vaccines being taken for small clinics. The absence of ice packs, thermometers and vaccine carriers resulted in high levels of wastage as leftover vaccines were discarded. Districts with more functional cold chains generally reported lower wastage. The exception was Sumkar distric. It had one of the best cold chains but reported vaccine wastage of 77%. Further work is needed to explore in detail the factors contributing to the high wastage rate in this district.

Interventions undertaken during outbreaks like the one in this study are often accompanied by a great sense of urgency. This may lead to rushed interventions that are poorly planned and coordinated. Future training should incorporate aspects of managing mass vaccination campaigns during an outbreak response. Regular audits of cold chain and an assessment of surge capacity for mass vaccination campaigns should be incorporated into routine activities.

Since this study was conducted in a single province, the results cannot be generalized to the country as a whole. Madang Province is a mountainous province with challenging health centre access; it is also one of the poorest provinces in Papua New Guinea. The assumption that only a small number of vaccine doses remained in the health centres after the campaign may have led to an overestimation of the wastage rate. Other limitations of this study included the focus on team leaders rather than all staff involved in the vaccination programme and the absence of on-site inspections. Depending on the size of the vaccination teams, there may have been variations in practice between the vaccinators that were not adequately captured in this study. A future study on vaccine wastage may focus on routine vaccination and an assessment of individual health workers, including on-site observations. A comprehensive training and audit plan that focuses on the routine vaccination programmes should be implemented. Systems should be developed to ensure the accurate documentation of routine vaccine administration and wastage at the provincial, district and health centre levels. This will assist not only in reducing wastage during routine programmes, but also in planning during outbreak response activities. Vaccine wastage report forms should be developed and routinely sent from the health centres to the District Health Office and from there to the Provincial Health Office. These vaccine wastage report forms should include reasons for wastage to guide ongoing efforts to reduce wastage.

An urgent and focused effort to strengthen the immunization programme in Madang Province would significantly reduce vaccine wastage and enhance the efficacy of both routine and outbreak response vaccination programmes. Efforts should focus on providing regular and pre-campaign training to vaccinators on correct technique, strengthening and monitoring cold chains and enhancing the documentation and evaluation of the immunization programme in the province. Enhanced documentation and improved supply management will prevent both stock-outs and excess wastage. Increasing efficiencies in the immunization programme by reducing wastage is critically important as the costs of routine and new vaccines continue to increase.
